# Intermediate MCAD Deficiency Associated with a Novel Mutation of the* ACADM* Gene: c.1052C>T

**DOI:** 10.1155/2015/532090

**Published:** 2015-12-22

**Authors:** Holli M. Drendel, Jason E. Pike, Katherine Schumacher, Karen Ouyang, Jing Wang, Mary Stuy, Stephen Dlouhy, Shaochun Bai

**Affiliations:** ^1^Division of Diagnostic Genomics, Department of Medical and Molecular Genetics, Indiana University School of Medicine, 975 West Walnut Street, Indianapolis, IN 46202, USA; ^2^Department of Molecular and Human Genetics, Baylor College of Medicine, One Baylor Plaza, Houston, TX 77030, USA

## Abstract

Medium-chain acyl-CoA dehydrogenase deficiency (MCADD) is an autosomal recessive disorder that leads to a defect in fatty acid oxidation.* ACADM* is the only candidate gene causing MCAD deficiency. A single nucleotide change, c.985A>G, occurring at exon 11 of the* ACADM* gene, is the most prevalent mutation. In this study, we report a Caucasian family with multiple MCADD individuals. DNA sequence analysis of the* ACADM* gene performed in this family revealed that two family members showing mild MCADD symptoms share the same novel change in exon 11, c.1052C>T, resulting in a threonine-to-isoleucine change. The replacement is a nonconservative amino acid change that occurs in the C-terminal all-alpha domain of the MCAD protein. Here we report the finding of a novel missense mutation, c.1052C>T (p.Thr326Ile), in the* ACADM* gene. To our knowledge, c.1052C>T has not been previously reported in the literature or in any of the current databases we utilize. We hypothesize that this particular mutation in combination with p.Lys304Glu results in an intermediate clinical phenotype of MCADD.

## 1. Introduction

Medium-chain acyl-CoA dehydrogenase deficiency (MCADD; OMIM 607008) is an autosomal recessive disorder resulting from a defect of mitochondrial *β*-oxidation of medium-chain fatty acids [1]. It is one of the most common inborn errors of metabolism with an incidence of 1 : 15,000 in 8.2 million newborns worldwide [2]. Metabolic demands that require more energy than available from glycogen stores, such as prolonged fasting, physical exercise, and intercurrent acute illness, may produce symptoms that if are unrecognized could potentially lead to metabolic crisis [3]. The clinical presentation of MCADD may vary from severe, such as hypoglycemia with seizures or death, to a milder form showing precursors of metabolic decompensation only. Intellectual disability and developmental delay are not common in this disorder if individuals are treated in a prospective manner; however, individuals with classic MCADD are at risk of losing developmental milestones after a metabolic event due to brain injury [1]. Furthermore, approximately 25% of individuals who are undiagnosed will die during the first metabolic crisis while anywhere from 30 to 40% will have some form of intellectual disability or developmental delay [4, 5].

*ACADM* (NM_000016.4), located at 1p31, is the only candidate gene causing MCAD deficiency. Mutations in the gene may result in reduced or abolished function of the MCAD enzymatic protein. c.985A>G, occurring at exon 11 of the* ACADM* gene is the most prevalent mutation in individuals of European descent. The single-base pair change causes the replacement of a lysine by a glutamate at position 304 of the mature protein, p.Lys304Glu. Approximately 81% of individuals presenting with MCADD will be homozygous for the p.Lys304Glu genotype, while another 18% will be heterozygous for p.Lys304Glu and an additional mutation [6]. Since the introduction of MCADD to the newborn screening (NBS) panel, new mutations continue to be identified. To date, besides the common mutation p.Lys304Glu, more than 90 mutations have been found in the* ACADM* gene (http://www.biobase-international.com/product/hgmd). These mutations are normally found in a compound heterozygous state with p.Lys304Glu and potentially lead to a loss of MCAD enzyme activity.

We report here, for the first time, a novel mutation in the* ACADM* gene, c.1052C>T.

## 2. Patients and Methods

**Figure 1:**
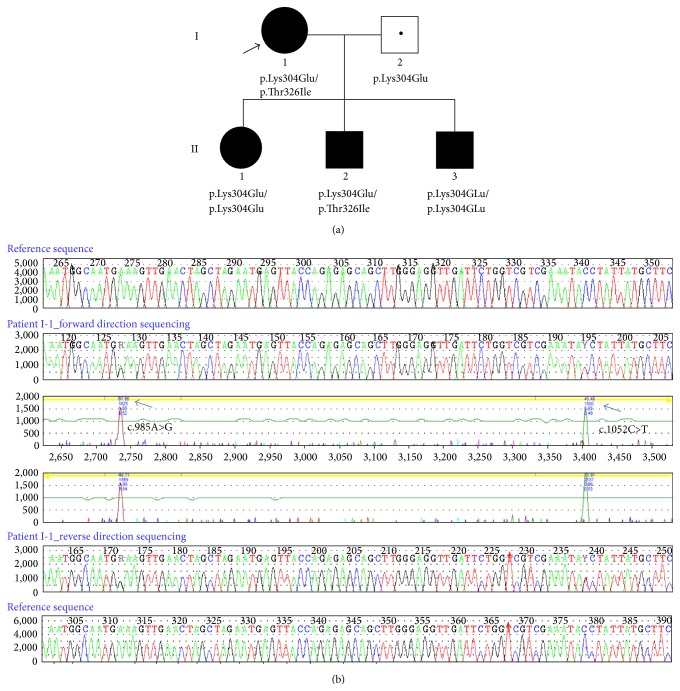
Novel MCADD mutation. (a) MCADD family pedigree. The proband (I-1) is indicated by a black arrow. The father (I-2) is a carrier as indicated by the dot in the center of the square. All affected individuals are indicated by filled circles (females) or squares (males). (b) DNA sequencing results.* ACADM* sequence analysis revealed a heterozygous sequence change of c.1052C>T, p.Thr326Ile in addition to the common mutation p.Lys304Glu in patient I-1. Blue arrows indicate the sequence change in patient I-1.

The proband, a 32-year-old Caucasian female (I-1; Figure 1(a)) with a positive MCADD newborn screening result in her first child, was diagnosed to have MCADD biochemically following her second pregnancy. She generally reports a very mild clinical phenotype with multiple, though minor, episodes of hypoglycemia as a child and adolescent. Her firstborn, a daughter (II-1), age 5 years, has a classic MCADD phenotype and, despite early NBS diagnosis and treatment with frequent feedings, L-carnitine and a low fat diet, has required multiple hospitalizations. The proband's second child, a son (II-2), now age 3 years, shares his mother's mild phenotype. The 1-month-old son (II-3) of the proband, also picked up on newborn screening, presented as a less severe phenotype compared to his sister, likely due to the dietary management and carnitine supplementation during the pregnancy. Their biological father (I-2) was also included as part of the family study (Figure 1(a)).

### 2.1. Sequencing Analysis

Genomic DNA was extracted from peripheral whole blood using a Qiagen Gentra Puregene blood kit (Qiagen, Valencia, CA, USA). All coding exons with exon-intron junctions of* ACADM* were PCR amplified and bidirectionally sequenced using BigDye Terminator v3.1 cycle sequencing (Life Technologies, Grand Island, NY, USA). Products were separated on an ABI PRISM 3130 XL genetic analyzer (Life Technologies, Grand Island, NY, USA). Sequencing data was analyzed by Mutation Surveyor software (SoftGenetics, State College, PA, USA).

### 2.2. Acylcarnitine Analysis

Plasma acylcarnitines were measured by tandem mass spectrometry [7] at Laboratory Corporation of America for patients I-1, II-2, and II-3. Analysis of patient II-1 acylcarnitine levels was performed at Duke University Hospital, Biochemical Genetics Laboratory.

## 3. Results and Discussion

Acylcarnitine patterns for MCADD patients display an increase in the levels of hexanoyl-CoA (C6), octanoyl-CoA (C8), decanoyl-CoA (C10), and decenoyl-CoA (C10:1) [8]. Accumulation of levels of C8 > 0.3 *μ*M is potentially considered diagnostic [9]. Plasma acylcarnitines were analyzed in the mother and her three affected children (Table 1). The 5-year-old daughter (II-1) had elevations of C8, C6, and C8/C10 ratios, with overall low carnitine. She was hospitalized in the prenatal intensive care unit at 5 days of age with breathing difficulties, hypoglycemia, and poor breast feeding. She developed elevated liver enzymes. Her symptoms were improved with intravenous glucose and regular infant formula. The proband had her carnitine levels checked at the beginning of the second trimester for her second child (II-2) (Table 1). She was provided carnitine supplementation for the duration of the pregnancy and was diagnosed biochemically by her acylcarnitine profile demonstrating an elevated C8 species. The second child (II-2) was formula fed. He had no metabolic decompensation. Following his abnormal NBS, plasma carnitine/acylcarnitine profiles were obtained and confirmed diagnostic for MCADD. The proband's third pregnancy began while on carnitine supplementation and was described as the easiest gestation of all three pregnancies, with better consistent nutritional intake and decreased nausea and vomiting. The third child was also identified on NBS and was biochemically diagnosed with MCADD (Table 1).

Sequencing analysis of the* ACADM* gene was performed in the proband (I-1). We detected a heterozygous c.985A>G, p.Lys304Glu mutation and a heterozygous c.1052C>T, p.Thr326Ile mutation (Figure 1(b)). Following these results, targeted mutation analysis of the three children was performed. The daughter (II-1) and the third male child (II-3) were found to be homozygous for p.Lys304Glu mutation. The girl's genotype correlates with her more severe phenotype. At birth, II-3 displayed a C8 profile similar to II-1 (Table 1), although slightly lower due to the mother being supplemented with carnitine for this pregnancy and not the first pregnancy. Patient II-2 was demonstrated to have the same genotype as I-1, p.Lys304Glu and p.Thr326Ile, consistent with his phenotype and the phenotype of his mother. Targeted sequencing analysis was performed for the father and indicated that he is a heterozygous carrier for the p.Lys304Glu mutation (Figure 1(a)).

To date, the p.Thr326Ile missense change has not been reported in MCADD patients. This replacement is a nonconservative amino acid change that occurs in the C-terminal all-alpha domain of the MCAD protein. This domain consists of densely packed *α*-helices, which appear to be important for the formation of a functional tetramer. Mutations located in this domain (including the common mutation p.Lys304Glu) were reported to affect helix-helix interactions and tetramer assembly and lead to aggregation and loss of function [10, 11]. Utilizing the SIFT Human Protein and Human Coding SNPs database (*Homo sapiens* GrCh37 Ensembl 63) [12, 13], we entered in the region of interest. The results of that analysis provided us with a prediction of a novel, nonsynonymous, and damaging change (SIFT score of 0.04). In addition to SIFT, we used the PolyPhen2 database [14] to determine if the amino acid change is damaging. The results from PolyPhen2 HumDiv and HumVar predicted that the p.Thr326Ile mutation is possibly damaging with a score of 0.922 and 0.623, respectively. Because both databases gave results in concordance with each other and the location in the protein is in the same C-terminal all-alpha domain as p.Lys304Glu, p.Arg309Lys, and p.Ile331Thr [11], it is suggestive that the p.Thr326Ile change is a deleterious mutation. Taken together, it suggests that this novel change is likely to be a disease causing mutation that is associated with a mild MCADD phenotype. It was reported that the mutations associated with the mild phenotype, such as p.Ala27Val, p.Tyr42His, and p.Arg309Lys, have a low risk of metabolic decompensation [15]. To determine the allele frequency of this novel mutation, a survey was performed in the MCADD patient database from Baylor College of Medicine. Five individuals heterozygous for p.Lys304Glu and p.Thr326ILe were identified among 500 patients. Additionally, a search of the ESP6500 database, 1000 Genomes database, and dbSNP concluded that this particular mutation has not been previously classified. Therefore, the allele frequency of p.Thr326Ile is less than 1%. To our knowledge, this is the first report of the c.1052C>T mutation that results in a mild MCADD phenotype.

## Figures and Tables

**Table 1:** Plasma concentration of C8 acylcarnitines in the proband and the three children.

Patient	Acylcarnitine profile C8 (*μ*M), first test	Acylcarnitine profile C8 (*μ*M), second test
I-1	3.2 (ref. range 0.02–0.30)	
II-1	13.3 (ref. range < 0.4)	0.36 (ref. range < 0.11)
II-2	4.23 (ref. range < 0.4)	6.76 (ref. range < 0.16)
II-3	9.7 (ref. range < 0.47)	3.58 (ref. range 0.02–0.31)
